# From Soft to Hard Biomimetic Materials: Tuning Micro/Nano-Architecture of Scaffolds for Tissue Regeneration

**DOI:** 10.3390/mi13050780

**Published:** 2022-05-16

**Authors:** Felicia Carotenuto, Sara Politi, Arsalan Ul Haq, Fabio De Matteis, Emanuela Tamburri, Maria Letizia Terranova, Laura Teodori, Alessandra Pasquo, Paolo Di Nardo

**Affiliations:** 1 Dipartimento di Scienze Cliniche e Medicina Traslazionale, Università Degli Studi di Roma “Tor Vergata”, Via Montpellier 1, 00133 Rome, Italy; arsalan.ulhaq@students.uniroma2.eu; 2 Department of Fusion and Technologies for Nuclear Safety and Security, Diagnostic and Metrology (FSN-TECFIS-DIM), ENEA, CR Frascati, 00044 Rome, Italy; sara.politi@uniroma2.it (S.P.); teodori@med.uniroma2.it (L.T.); alessandra.pasquo@enea.it (A.P.); 3 Centro di Ricerca Interdipartimentale di Medicina Rigenerativa (CIMER), Università Degli Studi di Roma “Tor Vergata”, Via Montpellier 1, 00133 Rome, Italy; fabio.dematteis@uniroma2.it (F.D.M.); emanuela.tamburri@uniroma2.it (E.T.); terranova@roma2.infn.it (M.L.T.); 4 Dipartimento di Scienze e Tecnologie Chimiche, Università Degli Studi di Roma “Tor Vergata”, Via della Ricerca Scientifica, 00133 Rome, Italy; 5 Dipartimento Ingegneria Industriale, Università Degli Studi di Roma “Tor Vergata”, Via del Politecnico, 00133 Roma, Italy

**Keywords:** micro/nanostructured biomaterials, tissue regeneration, architectural features, tissue engineering, cardiac muscular regeneration, cartilage regeneration, bone regeneration, scaffolding strategies

## Abstract

Failure of tissues and organs resulting from degenerative diseases or trauma has caused huge economic and health concerns around the world. Tissue engineering represents the only possibility to revert this scenario owing to its potential to regenerate or replace damaged tissues and organs. In a regeneration strategy, biomaterials play a key role promoting new tissue formation by providing adequate space for cell accommodation and appropriate biochemical and biophysical cues to support cell proliferation and differentiation. Among other physical cues, the architectural features of the biomaterial as a kind of instructive stimuli can influence cellular behaviors and guide cells towards a specific tissue organization. Thus, the optimization of biomaterial micro/nano architecture, through different manufacturing techniques, is a crucial strategy for a successful regenerative therapy. Over the last decades, many micro/nanostructured biomaterials have been developed to mimic the defined structure of ECM of various soft and hard tissues. This review intends to provide an overview of the relevant studies on micro/nanostructured scaffolds created for soft and hard tissue regeneration and highlights their biological effects, with a particular focus on striated muscle, cartilage, and bone tissue engineering applications.

## 1. Introduction

Due to lifestyle changes and an aging world population, the prevalence of degenerative diseases, cancers, and tissue and organ defects has dramatically increased. Consequently, there is a growing demand for organ replacements. However, the repair or substitution of organs and tissues remains a complex and unsolved issue. Current therapeutic approaches, such as surgery and transplantation, are limited by donor availability and immunological barriers and, therefore, novel treatment options are needed. Tissue engineering (TE), integrating information from various disciplines such as life sciences, material sciences, and engineering, holds the potential to reconstruct or to regenerate damaged tissues and organs by developing alternative therapeutic strategies [1,2].

In this field biomaterials are used to create a scaffold emulating the extracellular matrix that prompt tissue regeneration and/or new tissue formation through two primary TE strategies [3]. The first approach, known as “ex vivo TE”, is based on the building of a cell-laden scaffold outside the body, to obtain the formation of the desired tissue ”in vitro”. Subsequently, the construct is implanted into the injury site. In the second strategy, dubbed “in situ TE”, an acellular scaffold is directly transplanted into the site of damage where it can act as a “biophysical and biochemical cues delivery device” promoting endogen tissue regeneration [4]. The main advantage of this last strategy is that it does not involve the extensive manipulation of cells in-vitro to produce functional tissue, with the consequent risk of loss of native cellular phenotype [5], but uses external scaffold materials to induce the self-repair of damaged tissue [6].

However, the choice of the strategy is conditioned by both the regenerative capacity of the specific tissue target and the volumetric size of the damage. For example, in cardiac and nerve tissues, which have poor regenerative capacity, the “ex vivo TE” approach can provide better outcomes than the “in situ TE” approach, especially if the damage is severe and requires the replacement of a large portion of the tissue [4]. 

In all native tissues, cell behaviors are influenced by the surrounding dynamic microenvironment where the extracellular matrix (ECM) plays a crucial role [7]. Thus, the scaffold is designed to mimic the ECM of human tissues and to create a favorable microenvironment for the neo-tissue formation [1]. 

ECM is a three-dimensional multiscale network composed of an array of macromolecules, such as proteoglycan, collagen, elastin, and fibronectin, that provide structural and biochemical support to surrounding cells. Indeed, ECM contributes to the mechanical properties of tissues and works as a reservoir of anchored growth factors, cytokines, chemokines, and other bioactive molecules [8]. Cells and the surrounding ECM dynamically interact. Cells secrete, deposit, and remodel ECM to mediate its composition, mechanical properties, and topography. The ECM in turn transmits biochemical and physical signals to the cells to influence their phenotype and function [9]. Concerted biochemical and biophysical cues work together to dictate cell phenotype, gene expression, and finally cell fate.

Cells sense the physical cues of their surrounding environment through forces exerted on integrin-mediated adhesions which are translated into intracellular signals, in a complex and dynamic process called mechanotransduction, modulating several aspects of the cell behaviors [10]. The whole process includes physical and molecular events and results in cellular changes in order for the cell to adapt to the extracellular environment.

Cell–ECM interactions are triggered by integrins, transmembrane receptors which bind to ECM proteins (e.g., laminins, fibronectin) with their extracellular domain, and link to the cytoskeleton, with their cytoplasmatic portion. When integrins recognize and bind to ECM proteins, they rapidly associate with the actin cytoskeleton and cluster together, recruiting numerous proteins to form small focal complexes (diameter at nanoscale) located at the leading edge of membrane protrusions. Contractility-driven cytoskeletal tension of actomyosin induces maturation of nascent focal complexes into larger stable focal adhesions [11]. Focal adhesion transmits mechanical tension generated within cells across the plasma membrane to the external environment, thus providing a direct physical bridge and a biochemical link between cell and ECM [12,13].

Assembly, turnover, size, and distribution of these dynamic adhesions are affected by substrate stiffness and nanotopography [11].

Thus, cells can employ the direct physical interconnection between intracellular components and ECM to detect mechanical properties from their environment, such as ECM stiffness and topography, and consequently modulate their cytoskeletal organization and tension, morphology and, ultimately, cell function. 

Macromolecular compositions, mechanical properties, and structural architectures of the ECM are tissue specific. The type, concentration and organization of ECM components deeply affect the mechanical properties of tissue, such as stiffness. Stiffness, or rigidity, of a material is defined as the extent to which a material resists deformation in response to an applied force [14].

**Figure 1:**
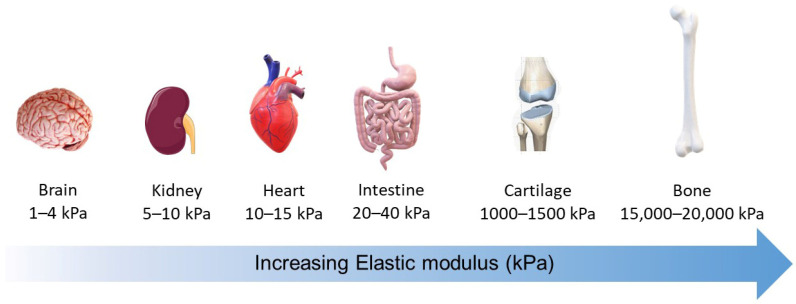
The stiffness of living tissues. The biomechanical properties of a tissue in terms of stiffness (elastic modulus), measured in pascals (Pa), vary between organs and tissues. Soft tissues such as the brain exhibit low stiffness, whereas tissues exposed to high mechanical loading, such as bone, exhibit elastic moduli with a stiffness that is several orders of magnitude greater. Values of Young’s modulus were from ref. [14]. The figure was created using Servier Medical Art and 3d models of Microsoft 365.

The different tissues exhibit various degrees of stiffness (defined as Young’s modulus, or elasticity, of a material). Neural tissues, as well as most abdominal organs, are generally characterized as soft tissues. Tissues exposed to high mechanical loading, such as bone, exhibit elastic moduli with a stiffness that is several orders of magnitude greater. Cartilage, ligament, tendon, and bone are the stiffest tissues of the human body and are usually indicated as hard tissues [15] (Figure 1). Materials used as matrices for cellular study range from hard glass (65 GPa), to thermoplastic polymers such as polystyrene (2.3 GPa) and poly(lactic-co-glycolic acid) (1.31 GPa for PLGA 50/50), to elastomeric polymers such as polydimethylsiloxane (PDMS; 3.4 MPa), and to soft hydrogels (from several pascals to several kilopascals) [11,15].

The native ECMs of tissues are organized in hierarchical structures consisting of micro- and nanoscale topographic patterns that are essential to tissue-specific function and its mechanical properties [16]. For example, the complex cardiac tissue architecture is maintained by wide three-dimensional ECM networks composed of collagen (fibrils with diameter range from 100 to 300 nm), elastin bundles (fibrils of ~0.2 mμ thickness), and interconnected basement membranes. This ECM network orients the cardiomyocytes, mechanically couples them to each other and to neighboring capillaries and nerves, and provides elastic support during ventricular contraction. The helical wrapping of the laminar structures generates an anisotropic syncytium that endows the cardiac muscle with unique electrical and mechanical properties [17].

**Figure 2:**
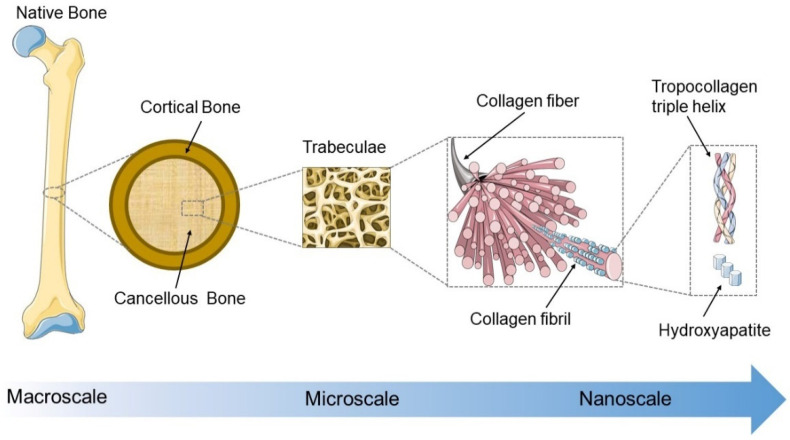
The hierarchical structure of natural bone from macro to nanoscale. The schematic representation of the long bone structure is shown as an example. In the macroscopic view, bone cross-section can be divided into an external and an internal part. The bone external structure consists of compact cortical bone which is composed of densely packed cylindrical osteons. The internal cancellous bone has a porous trabecular structure. Osteons and trabeculae are made up of lamellae with different collagen fiber patterns. The collagen fibers (~1 µm) are composed of bundles of mineralized collagen fibrils (~100 nm), in which hydroxyapatite nanocrystals (nanoscale size) are deposited in the gaps between collagen molecules (tropocollagen triple helices). The figure was created using Servier Medical Art.

The bone tissue possesses a unique combination of remarkable strength and toughness due to excellent mechanical properties which are closely associated with its ECM hierarchical structures and precise organization of the inorganic (mostly carbonated hydroxyapatite) and organic phases (mainly type I collagen) [18]. In the macroscopic view, natural bone consists of compact cortical bone and trabecular cancellous bone, which are composed of lamellae with different collagen fiber patterns. The collagen fibers (~1 μm) are composed of bundles of mineralized collagen fibrils (~100 nm), and hydroxyapatite nanocrystals are deposited in the gaps between collagen molecules [19,20] (Figure 2).

Taking inspiration from biochemical, mechanical and structural information present in native ECMs, biomaterials of the scaffold should be designed to provide an artificial milieu with micro and nanoscale structures found in natural tissues, which guide cells for re-growing or regenerating damaged tissues [21,22]. In recent years, advances in scaffold production techniques for tissue engineering purposes provided the possibility to accurately create 3D scaffolds with defined nano and microscale architectures [23,24,25].

The architectonic features include porosity, fiber orientation, and surface topographies, which can regulate cell behaviors, such as adhesion, migration, proliferation, and differentiation, and thereby influence tissue formation.

The materials used to create scaffolding have various origins, and specific chemical compositions, and physical and mechanical characteristics, which may be properly modified to match those of the target tissue [26].

Combinations of two or more types of materials were often developed with the aim of increasing the scaffold properties and to improve tissue interaction. The resulting composite or hybrid scaffolds show unique characteristics required for different applications in hard and soft tissue engineering [21,27]. 

This review offers an overview of the recent micro-nano structured scaffolds and highlights their biological effects. We first highpoint the importance of the scaffold micro/nanostructure on cell behaviors. Next, we review the main materials and scaffold processing techniques. Then, some relevant examples of application of micro/ nanostructured scaffolds for striated muscle, cartilage and bone TE are described. Finally, some limitations and future challenges are briefly discussed.

## 2. Impact of Micro/Nano Architectural Features of Scaffolds on Cell Behaviors

**Figure 3:**
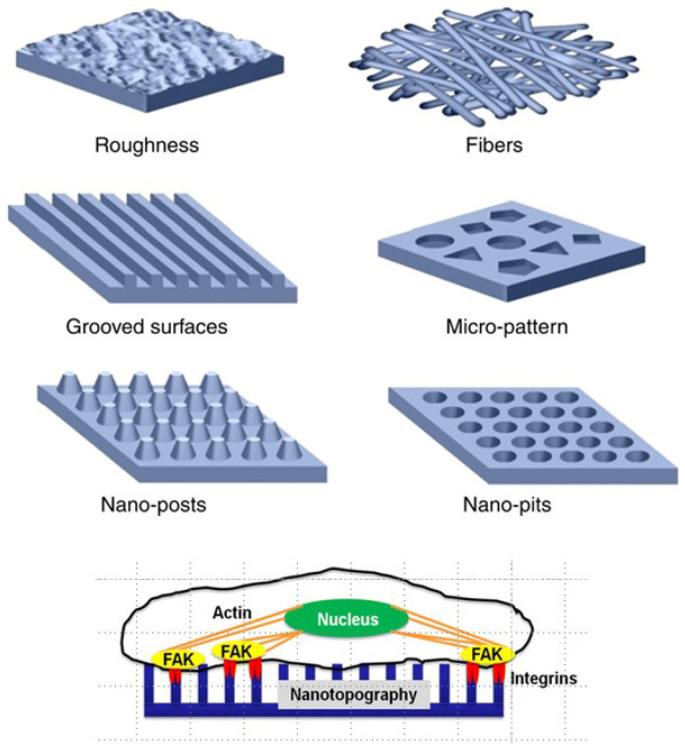
Schematic representation of micro/nanoscale surface patterns (Reprinted with permission from ref. [20] Copyright 2022, Elsevier and schematic representation of cell–nanotopography interactions. Adapted with permission from ref. [29] Copyright 2022 American Chemical Society.

The architectonic features of the scaffold, such as porosity, fiber orientation, and surface topographies, play an essential role in guiding cell phenotype, organization, and resulting tissue formation (Figure 3). The size, shape, and orientation of these features can regulate cell adhesion, morphology, alignment, migration, proliferation, and gene expression [12,28].

Among the geometrical cues provided by the intrinsic architecture, porosity is one of the major factors for the growth of new tissues and their reorganization. Generally, scaffolds should be porous to facilitate cell infiltration, oxygen diffusion, nutrient transport, and vascularization [30,31]. However, excessive porosity could imply poor mechanical properties [30]. The minimal pore size for scaffold material was reported as approximately 100 µm, which allows cell infiltration, considering that most mammalian cells are between 10 and 100 µm in diameter [32]. The ideal pore size depends on the cell type of specific tissues [33,34]. Generally, however, the pore size should be in a range that facilitates cell penetration and migration during cell seeding, nutrient diffusion, and removal of metabolic substances. Tiny pores could obstruct cellular penetration, ECM deposition, and neovascularization; however, small pores offer more adsorption sites for protein and other bioactive molecules, and promote cell adhesion [35]. Other studies indicated the larger pores (between 100 and 600 µm) induce better growth and vascularization of various tissues, after the scaffold implant. On the other hand, extremely large pores (more than 500–600 µm) could decrease cell adhesions and excessively increase the rate of degradation of the scaffold [36,37]. Well-interconnected pore structures are crucial to facilitate cell infiltration, nutrient supply, and waste removal [38]. Porosity, interconnectivity, pore size distribution, and morphology of the pores, also play an important role in determining the ultimate mechanical properties of the scaffold, such as its elasticity and degradation rate [30]. An accurate balance among all these parameters is required to obtain a scaffold with appropriate mechanical and biological properties, which provides an ideal microenvironment for influencing cell fate.

Over recent decades, a significant amount of work was reported on the study of cell response to a variety of micro/nanotopographical features, such as roughness, micropattern, nanopits, nanogrooves, etc., (Figure 3). Some of these topographical features can be fabricated with different distributions or orientations (aligned, ordered, and disordered). Cues for contact guidance can orient cells along the direction of anisotropic features, such as ridges, grooves, and gratings or parallel single fibers [12]. Aligned cues in the form of nanogratings were sufficient to direct cell adhesion, elongation, alignment, and neurogenic differentiation of human MSCs. A contact guiding mechanism was a consequence of the alignment and elongation of focal adhesions [29]. Single cells on aligned patterns have a much higher alignment ratio compared with gridded and unpatterned substrates [39]. Furthermore, mesoscale cues (>100 µm) in combination with microscale cues (1–2 µm) acted synergistically to enhance the alignment of MSCs when these cues were in the same direction. However, the mesoscale confinement cues overrule the microscale cues when presented perpendicularly to each other [12,39].

These studies have revealed that the microtopography (feature size larger than 10 µm, comparable in size to the cell body) mainly has effects on whole cell morphology, while the nanotopography is involved particularly with subcellular-sensing mechanisms and involves physical interaction between cell molecules (e.g., integrin receptors) and nanoscale geometric cues [28]. Nanoscale structural stimuli influence cell adhesion, morphology, proliferation, and differentiation [40,41]. Cells sense nanoscale geometric cues from their environment through small (diameter at nanoscale) cell membrane projections (filopodia) that form nascent focal complexes. Such cues may represent differences in molecular conformation, surface topography or roughness, fiber diameter, and other parameters that can directly influence the integrin clustering and, thus, focal adhesion maturation and their resultant size and distribution [42]. 

Although many studies have been produced on the cellular response to different topographical features as relief structures in 2D substrates, it is still difficult to introduce these topographic models into 3D scaffolds [43]. However, advances in additive-manufacturing technologies and the combination of various materials and techniques seem to be optimal strategies to produce functional scaffolds with defined architectures and surface topography.

## 3. Materials for Micro/Nano-Structured Scaffold Fabrication

Material selection plays an important part in the design and production of scaffolds for tissue regeneration applications. A biomaterial can be defined as any material used to produce devices that can replace a part or function of an organism in a safe, economical and physically plausible way [44].

Based on their chemical composition, biomaterials used for scaffold fabrication are generally classified into polymers, inorganic biomaterials (metals and ceramics), and composites. According to their origin, biomaterials can be natural or synthetic [31]. A broad variety of naturally derived and synthetic-based polymers have been applied for scaffold processing. 

Natural polymers are obtained from renewable sources, such as animals, algae, plants, and microorganisms. The most popular natural polymers used for scaffold fabrication include polysaccharides (e.g., chitin, chitosan, alginate, hyaluronic acid, cellulose, agarose, dextran) and proteins (e.g., collagen, fibrin, fibrinogen, gelatin, silk, elastin, myosin, keratin, and actin) [3]. The main advantages of these materials comprise biocompatibility and bioactive behavior. Nevertheless, natural materials suffer from several limitations, such as low reproducibility and limited control over the mechanical properties of the fabricated scaffold [22].

Synthetic polymers are produced from hydrocarbon building blocks in a laboratory setting. The most widely studied synthetic polymers include poly(L-lactic acid) (PLLA), polycaprolactone (PCL), poly (lac-tic-co-glycolic acid) (PLGA) polyethylene glycol (PEG), and polyurethane [45]. Compared with natural polymers, synthetic polymers with their greater processing flexibility and controllable physical and mechanical properties are the predominant scaffolding materials. They can be used to tailor both soft and hard tissues. However, the synthetic polymers lack cell adhesion sites and require chemical modifications to enhance cell attachment and biocompatibility [46,47].

An important class of polymers is represented by hydrogels which are probably the most widely explored type of material in TE, due to their biocompatibility, biodegradability, and extracellular matrix-mimicking ability. Hydrogels are made of hydrophilic polymers rich in polar moieties, such as carboxyl, amide, amino, and hydroxyl groups, held together by chemical bounds or physical intra-molecular and inter-molecular attractions. Their main feature is the ability to absorb enormous amounts of water or biological fluids and swell without dissolving [48]. Hydrogels can encapsulate cells and can be loaded with growth factors, or other bioactive molecules, that are essential for the promotion of cell differentiation. Recently, considerable interest was drawn to ‘smart hydrogels’, which are known to have the ability to respond to changes in their external environment [49]. Composites or blending of synthetic and natural polymers can provide a variety of physicochemical and biological characteristics [3].

Inorganic biomaterials comprise metals (e.g., titanium and its alloys, gold, silver) and bioceramics (e.g., calcium phosphates including hydroxyapatite, calcium carbonates, bioactive glasses, alumina, and zirconia). Inorganic biomaterials such as titanium and its alloys, alumina and zirconia are usually defined as “bioinert materials” because they have no interaction with the adjacent tissue after implantation, typically being applied as structural-support implants, such as bone devices and femoral heads [50]. On the other hand, inorganic biomaterials can act as bioactive materials promoting tissue regenerative processes. For example, mineral-based biomaterials can release bioactive ions (e.g., Ca, Mg, Cu, Zn) which influence cell response or modulate the immune microenvironment and tissue regenerative processes [51].

The addition of inorganic materials to polymeric scaffold composition can enhance many resulting biophysical characteristics (e.g., topography, charge, electrical conductivity, and stiffness), as well as biological properties (e.g., cell–matrix interactions, regulation of cellular response, and antibacterial activity) [22]. For this reason, various inorganic materials are often dispersed or incorporated in organic matrices, improving their functional properties.

Based on a material’s size, nanomaterials can be defined as materials (organic or inorganic) possessing, at minimum, one external dimension in the range of 1–100 nm [52]. They may have different shapes, such as nanoparticles, nanowires, nanolayers, nanofibers, and nanotubes, and when incorporated into the scaffolds they strongly influence the resultant mechanical proprieties and nano-topography. Since components of ECM, such as biological molecules, are nanoscaled structures, cells are programmed to interact with nano-sized materials, which can affect cell behavior [53].

Generally, for tissues, such as skin, brain, and muscle, soft materials that can easily be deformed by thermal fluctuations and external forces are preferred due to their low elastic moduli. Soft materials include gels and hydrogels, elastomers, and polymers. Materials with higher elastic moduli that cannot be compressed are known as hard materials and are generally used for bone TE. These materials comprise inorganic biomaterials, such as zirconia, titanium and bioceramics [54].

However, in scaffolding strategies, multiple types of biomaterials with distinct properties are often combined and fabricated to be tailored to a particular application in soft or hard TE. The combination of two or more materials with different properties can improve the characteristics and functionality of the resulting construct. The range of scaffolds produced for in tissue regeneration includes, porous, fibrous, hydrogel, and 3D-printed scaffolds. The fabrication of these scaffolds can be obtained through different techniques. In the following sections, we describe the main fabrication techniques.

## 4. Main Manufacturing Methods for Micro/Nano-Structured Biomaterials

A large number of technological approaches were applied for the purpose of creating efficient biomimetic micro/nano-structured biomaterials for each specific tissue type. Traditional biomaterial fabrication methods include salt leaching, gas foaming, and the lyophilization technique, which were usually applied to produce porous scaffolds. In the salt leaching method, pore sizes are controlled through the use of porogens, such as wax, salt, and sugars. In the gas foaming technique, a porous structure is produced by using high-pressure carbon dioxide and controlling the amount of gas. Finally, in the lyophilization technique, scaffolds are designed via the sublimation of the desired concentration of a solution [20,31]. Thermally induced phase separation is often used to produce porous scaffolds, while electrospinning generates micro- or nanofibers. With advancements in technology, some innovative methods, such as additive manufacturing (AM) technologies and self-assembly, have been widely applied to produce novel biomaterial scaffolds for soft and hard TE [55]. The most common methods are briefly described below.

### 4.1. Thermally Induced Phase Separation

Among the possible techniques to produce porous scaffolds, the thermally induced phase separation (TIPS) method is widely used for scaffold fabrication due to the possibility of obtaining a well-interconnected polymer network with a wide range of pore sizes, through an easy-to-tune, fast, and adaptable process. In this procedure, a change in temperature induces a homogeneous polymer solution to be separated into two distinct phases, a polymer-rich and a solvent-rich phase. After solvent removal by extraction, evaporation or sublimation, the polymer-rich phase is converted into the skeleton of a porous scaffold, while the removal of the solvent creates the final porosity [56].

For example, highly porous 3D PLLA scaffolds have been prepared by directional thermally induced phase separation (dTIPS) starting from 1,4-dioxane/PLLA solutions. The processing parameters were optimized to achieve an overall porosity for the 3D scaffolds of about 93%, with a degree of interconnectivity of 91%, ensuring high perfusion. The resulting pore network formed branched canals strongly resembling the vascular patterns. The multiscaled porous architecture of the produced scaffolds allowed the growth of bone marrow stromal cells (BMSCs) across the entire scaffold thickness (approximately 1 mm) and preserved their multi-potency towards differentiation after a long-term culture [57]. In recent years, advancements of the TIPS technique enabled the fabrication of a variety of architectures and pore morphologies in micro/nanometer scale in different TE applications of soft and hard tissues [58]. For example, PLLA can be mixed with chitosan, a natural polymer, to fabricate scaffolds that support nerve cells for neural TE applications [59] or with hydroxyapatite to create a porous scaffold for bone tissue regeneration [60]. Thin, porous scaffolds were produced via TIPS for use in dermal wound repair, using ethylene carbonate as the solvent, to encapsulate a poly (lactic-co-glycolic acid) (PLGA) mesh into a porous PLGA network. These biphasic scaffolds exhibited a high tensile strength, porosity of 94%, and good biocompatibility and biodegradability [61].

To simulate the biomechanical properties and the anisotropic architecture of native heart muscle, an anisotropic and biodegradable polyurethane porous scaffold was fabricated via TIPS using various polyurethane types and concentrations. The polyurethane porous scaffold was combined with a porcine myocardium-derived hydrogel to form a biocompatible and bioactive biohybrid scaffold [62]. Different architectures can be obtained through the manipulation of the TIPS process conditions or combining TIPS with other techniques [58].

### 4.2. Sol–Gel Method

This method is commonly used to synthesize bioceramics and bioactive glasses. The process consists of the following steps: first, the precursors (inorganic or organic metal compounds) are mixed with water to form sols after hydrolysis and condensation reactions; second, the sols are foamed, and start to condense after the addition of surfactants and catalysts; third, the foamed sols are transferred to a mold, where a gel is formed. The final thermal treatment then densifies the matrix [20,63].

### 4.3. Electrospinning

Electrospinning involves a process governed by electrohydrodynamic phenomena [12], which allows the fabrication of micro- or nanofibers [64]. A standard electrospinning system consists of a syringe with a needle, a high-voltage power supply, and an electrically conductive collector. In the first step of the process, a polymeric solution is loaded in the syringe. To create the force of extrusion, a high voltage is applied to the needle of the syringe and the collector. Due to the potential difference generated, the particles within the solution are charged, thereby creating a repulsive force. At a critical voltage, the repulsive force overcomes the surface tension of the solution, and a jet erupts from the needle of the syringe [65]. Once the electric field reaches a critical value at which the repulsive electric force overcomes the surface tension, the polymeric solution is ejected from the syringe needle. The fibers are formed during the fast evaporation of the solvent and are deposited onto the collector [47]. The technique may be suitable and combined with other methods for generating aligned fibers which can play a key role in guiding cell alignment and elongation [66]. For example, a cell-laden hierarchical scaffold was developed by adapting the electrospinning method with parallel electrodes to align the PCL micro/nanofibers deposited on perpendicular PCL struts. Finally, a bioink containing C2C12 myoblasts was printed onto the fabricated PCL structure. In this hierarchical scaffold, the PCL-fiber alignment strongly induced cell orientation and differentiation, leading to the formation of myotubes [66].

### 4.4. Soft Lithography

This method includes a set of fabrication techniques that uses elastomeric stamps, molds, and conformable photomasks for patterning two- and three-dimensional structures ranging from micrometer to nanometer scale [67]. These techniques are used for micro/nanopatterning structures and their common characteristic is that they involve fabrication or replication of a “master”. This master can either be used as a stamp or as a mold to pattern an elastomer, which is later cross-linked. After cross-linking, the patterned elastomer is applied like a stamp/mold for the micro/nanopatterning of other material [68]. The benefits of soft lithography include not only a relatively lower cost, easier setup, and high throughput, but also a pattern resolution ranging from nanometer to micrometer precision. One drawback of soft-lithography is the need to utilize another lithography method, such as photolithography or e-beam lithography, to fabricate the stamp master. However, these steps only need to be done once as the master can be used repeatedly to produce the stamps

### 4.5. Additive Manufacturing Technologies 

Additive manufacturing (AM) is a technology that creates three-dimensional objects from digital data (3D model) by depositing materials in a layer-by-layer controlled manner. Various methods and printing materials are employed in AM, such as fused deposition modeling (FDM), stereolithography (SLA), projection microstereolithography, selective laser sintering (SLS), multiphoton lithography, and 3D printing.

FUSED DEPOSITION MODELING (FDM) uses a movable head to create a 3D object by depositing a thread of molten thermoplastic polymer above a build platform. Processed material, in a semi-liquid state, is added layer by layer. Each cross section is formed due to print-head movement (in the X and Y axes) according to the CAD design. FDM has good efficiency, easy material replacement, and low costs of operation and implementation. The main advantage of FDM over 3DPrinting is that it does not require any organic solvent, and there is no need to remove excessive polymer powder. FDM also has several limitations, such as narrow selection of biomedical materials that are possible to process [69].

STEREOLITHOGRAPHY (SLA) is a rapid prototyping technique that uses photopolymerization to fabricate 3D scaffolds layer by layer, according to a computer design program. In SLA, a photosensitive liquid resin is irradiated by a UV light beam and allowed to deposit and solidify over a moveable platform, forming the first layer. Once the first layer has solidified, the platform is lowered and the process is repeated for several layers until the desired prototype is obtained [70].

PROJECTION MICROSTEREOLITHOGRAPHY is a technique based on a manufacturing principle very similar to stereolithography but implements process improvements that result in a far better resolution (up to 5–10 mm resolution) allowing the fabrication of 3D components by layer-by-layer curing of a photopolymerizable material [71]. Such resolution would enable the fabrication of scaffold-accurate geometries with high porosity, large surface area, and pores of appropriate size for cell proliferation [72].

SELECTIVE LASER SINTERING (SLS) uses energy provided by the laser to melt and fuse the powders and then stack them layer by layer to form a printed part based on 3D model data [73]. The advantages of this method are recyclability of unused powder, 20–150 µm printing resolution, and excellent mechanical properties of the SLS bioprints. However, the high operative temperatures, due to laser radiation, limit the manufacturing of natural polymers and the encapsulation of biomolecules or cells [69].

MULTIPHOTON LITHOGRAPHY, also known as direct laser lithography or direct laser writing (DLW), allows the formation of 3D structures from photosensitive materials at the micro and nanoscale, down to resolutions as low as sub-100 nm [74]. The technique works via nonlinear absorption of two or more photons by photosensitive monomers and the resulting local polymerization. Typically, DLW involves focusing an ultra-fast laser beam into a small volume inside a photosensitive resin to initiate the local polymerization. The construction of the scaffold occurs by moving the laser beam according to a path representing a CAD model. Direct laser writing offers excellent fabrication of 3D micro- and nanostructures with fine resolution, as well as high writing speed. However, this technique requires expensive and specialized equipment [75].

3D PRINTING is an additive manufacturing technique that enables the fabrication with high precision through a layer-by-layer building process of tissue-like constructs, replicating the complex architecture of biological systems. The outcome of this process results in the development of three-dimensional scaffolds with well-defined topographical properties. In this procedure, the printing materials, or “ink”, are used to print acellular structures that are subsequently populated with cells or are directly transplanted to the site of damage [76]. In the 3D-bioprinting process, the material being printed, called “bioink”, typically includes biomaterials, live cells, and/or bioactive factors. Bioinks are often formulations of hydrogel precursors that can provide a highly hydrated environment for the encapsulated cells [77]. A typical 3D-bioprinting process begins by forming an organ blueprint from a set of images obtained from medical imaging technologies, such as computer tomography (CT) or magnetic resonance imaging (MRI); this information is converted into standard model library (STL) direct-instruction software for printing hardware [78]. Bioprinting technologies can be divided into three main categories: extrusion, droplet, and laser-based bioprinting. Extrusion-based bioprinting (EBB) employs mechanical, pneumatic or solenoid dispenser systems to deposit bioinks in a continuous form of filaments, to form 3D scaffold structures. Droplet-based bioprinting relies on the generation of bioink droplets by thermal, acoustic, or electrical stimulation. Laser-based bioprinting uses a laser as an energy source to deposit biomaterials onto a substrate to create 3D print structures by a photopolymerization principle. Among these, the EBB, also known as direct ink writing, is the most widely used approach to 3D bioprinting due to its cost-effectiveness and versatility [79].

Over recent years, significant advancements were made in integrating secondary techniques accompanying the modalities of bioprinting. For example, sacrificial bioprinting was developed to produce tissue blocks encapsulating interconnected hollow channels simulating the vascular network [80]. The development of methods such as “freeform reversible embedding of suspended hydrogels” (FRESH), has enabled the 3D printing of soft biomaterials within a thermoreversible hydrogel support bath; this support gel solidify around the extruded 3D structure, preventing it from collapsing due to gravity during the bioink deposition process [81]. In addition, the development of bioprinters, equipped with multiple pressure controllers and dispensing heads which extrude cell-laden hydrogels, together with different biomaterials, boosted the capacity to build complex tissues [82].

## 5. Applications of Micro/Nano-Structured Materials for Soft and Hard Tissue Regeneration

In this section, representative examples of micro-nanostructured biomaterials in vitro and tissue generation in vivo are described based on the tissue-specific environment.

### 5.1. Micro/Nano-Structured Scaffolds for Cardiac Muscle Regeneration

The heart is a vital organ that continuously functions as a muscle pump to push blood through the body’s tissues every day. The heart muscle, at least in mammals, has little regenerative potential after injury, such as myocardial infarction (MI); this frequently leads to irreversible cardiomyocyte (CMs) loss and scar formation, and finally to heart failure [83]. The current therapeutic options for patients after cardiac infarction are limited. Consequently, the design of the novel therapies based on biomaterials could be useful for cardiac regeneration [84,85].

The cardiac muscle is considered to be a composite, anisotropic, viscoelastic material made of various cell types, such as CMs, pericytes, endothelial cells, nerve cells, and fibroblasts surrounded by an ECM network. The cardiac ECM is composed mainly of collagen and elastin bundles embedded in a proteoglycans-based interfibrillar matrix [18]. The ECM network maintains the mechanical continuity between myocytes ensuring the electrical connectivity and provides elastic support during ventricular filling. 

Collagen is the most abundant protein in the heart and confers strength and structural integrity. Collagen I forms rod-like fibrils with a diameter of ~300 nm, while collagen III forms smaller fibrils with a diameter of ~100 nm [86]. Elastin forms unit fibrils of ~0.2 µm thickness that confer elasticity [87]. Hence, to engineer the tissue of heart, which beats cyclically and constantly throughout life, the biomaterial should be as soft and elastic as heart muscle [88]. Moreover, the hierarchical structure of heart muscle is highly ordered, from the helical and weaving arrangement of myocardial fibers in the ventricles to the parallel alignment of myofibrils in individual CMs [89], which are the contractile cells occupying most of the volume of the heart (approximately 70–85%) [90]. 

Thus, an ideal scaffolding strategy would require primarily a source of functional CMs. In the past, several cell types were indicated as potentially able to regenerate the myocardium, such as bone marrow-derived mononuclear cells, MSCs, hematopoietic stem cells, endothelial progenitor cells, cardiac progenitor cells, and pluripotent stem cells (PSCs) [91]. Among these cells, pluripotent stem cells (PSCs) have displayed a greater potential to generate differentiated CMs and to functionally remuscularize the infarcted heart of preclinical models, including that of macaque monkeys [92,93]. In particular, CMs derived from induced PSC (iPSC) have emerged as a leading candidate for cardiac tissue reconstruction and for future applications in the clinical setting [94]. However, PSC-derived CMs are more similar to fetal rather than adult CMs and effort is needed to implement differentiation protocols to generate a pure population of mature cardiomyocytes [95]. In the native heart, the CMs are aligned into anisotropic ECM and electrically coupled to each other. CMs are constantly active, stimulated to beat and, therefore, have a high metabolic demand for oxygen. Consequently, cardiac scaffolds are, in general, highly porous and perfusable to enable oxygen supply in vitro and promote angiogenesis after transplantation [31]. In recent years, the studies aimed at rethinking one or more structural features of the heart tissue, such as porosity and aligned architectures.

For example, porous nanofibrous PLLA scaffolds were prepared by combining a unique phase-separation procedure with a sugar-sphere template leaching process [96]. The PLLA scaffolds exhibited a uniform porous structure and a high level of interconnectivity among the pores. The walls of the pores consisted of nanofibers with an average diameter between 100 and 200 nm, mimicking nanofiber features of natural collagen fibers in cardiac ECM. These nanofibrous PLLA scaffolds supported cardiovascular progenitor cell commitment into cardiomyocyte, smooth muscle, and endothelial cells, in vitro and in vivo. The influence of micropore geometry on murine cardiac progenitor cell behaviors was studied in biocompatible scaffolds with different chemical compositions and micro-architectures [97]. PLLA, PCL, PLGA, and PCL/PLLA (BLEND) polymers were used to fabricate 3D microporous scaffolds with square (diameter: 100 mm) or hexagonal (diameter: 134 mm) pores through a layer-by-layer “pressure-assisted microfabrication” (PAM) technique. A mathematical model developed in this study predicted the anisotropic distribution of the elastic modulus in the pore neighborhood of the 3D constructs. All the scaffolds supported murine cardiac progenitor cell adhesion and proliferation, but only the square PLLA 3D scaffold strongly influenced cell alignment and differentiation, enhancing the expression of CM-specific proteins. It is noteworthy that cardiac cells aligned in a characteristic direction on the square PLLA 3D scaffold, corresponding to the areas theoretically endowed with minor stiffness that approximates the myocardial tissue. 

Similarly, square-grid scaffolds made of polyurethane promoted proliferation and enhanced expression of cardiomyocyte differentiation markers in human cardiac progenitor cells. These scaffolds were prepared by melt–extrusion additive manufacturing and functionalized with laminin-1 [98]. Polyurethane-based elastomers have been often employed for the cardiac scaffold production due to their tunable elasticity [99]. 

Xu et al. recently used various polyurethanes with different soft segments to fabricate, via the TIPS method, an optimal porous anisotropic scaffold with mechanical properties very similar to those of the myocardium. To improve the bioactivity of the structure, the porous scaffold was combined with a porcine myocardium-derived hydrogel to form a resultant biohybrid scaffold, which displayed excellent mechanical properties and allowed good cellular infiltration, after rat subcutaneous implantation [100].

An accurate reproduction of the cardiac structures can be obtained by using approaches of 3D printing based on photo-polymerization [101]. Among these techniques, microstereolithography allows for precise manufacture of 3D cardiac scaffolds using polymers, such as polyethylene glycol diacrylate (PEGDA). To better mimic the cardiac ECM microenvironment, a hybrid 3D construct was created based on a “scaffold-in-scaffold” design. This construct was composed of a porous printed PEGDa woodpile which was embedded into a softer PEGDa hydrogel. Human cardiac progenitor cells encapsulated into the PEGDa hydrogel, differentiated into cardiomyocytes, which aligned in an orderly manner forming a multilayered tissue. However, robust cardiomyocyte maturation, such as distinct sarcomeric organisation was not achieved [102]. 

A cardiac muscle patch with a native-like cardiac ECM architecture was generated using multiphoton lithography and gelatin methacrylate. hiPSC-derived CMs, endothelial cells, and smooth muscle cells were seeded onto the scaffold and incubated to create the engineered cardiac tissue. After 7 days, human iPSC-CMs exhibited functional maturation, alignment, and elongation of cells within the channels of the scaffold. In addition, these constructs exhibited synchronous beating. When tested in mice with myocardial infarction, this muscle patch improved cardiac function and reduced infarct size after 4 weeks [103]. To mimic the anisotropic architecture of the myocardium, a microstructured PLGA/gelatin scaffold was fabricated by using soft lithography [104]. The scaffold was obtained by depositing a PLGA/gelatin blend onto a silicon mold. On its surface, this mold had micropatterning with a predefined geometry, based on the morphological analysis of a decellularized swine cardiac tissue. The layers of the scaffold presented channels and reliefs of different sized scales, ranging from 30 µm to 500 µm. This PLGA/gelatin scaffold promoted adhesion, elongation, ordered disposition, and early myocardial commitment of human mesenchymal stem cells without additional stimuli, such as differentiating factors. Similar PLGA/gelatin constructs were further enhanced by functionalization, with adenosine acting as a cardioprotective factor [105]. 

Capillary lithography-based approaches are able to fabricate arrays at the nanoscale level with high precision. In a key study, scalable PEG hydrogel arrays were fabricated to mimic the myocardial ECM ultrastructure [106]. The nanofabricated substrates were formed from parallel nanogrooves and nanoridges with different size characteristics (width of the grove and ridge and height). Nanopatterned substrates were reported to guide CMs alignment, resulting in enhanced expression of Cx43, anisotropic propagation of action potential, and contractility characteristics of the native heart architecture.

Electrospinning methods can generate fibrous scaffolds with alignment cues to guide the development of myogenic or cardiac precursors into 3D muscle grafts. Electrospun PCL-collagen nanofibers of these grafts induced parallel alignment and increased myogenic differentiation in rat MSCs with or without growth factors or co-cultivation with myoblasts [107]. Importantly, a more recent study has demonstrated that aligned electrospun PCL-collagen-nanofibers could increase the myogenesis of MSCs upon co-cultivation with myoblasts, even in the absence of serum [108]. Zang’s research group fabricated a PLLA/chitosan scaffold by conventional electrospinning and demonstrated that scaffolds with aligned fibers could increase cardiomyocyte viability, elicit cell elongation, and enhance production of sarcomeric α-actinin and troponin I, with respect to scaffolds with randomly oriented fibers [109]. Gosh et al. suggested that aligned nanofibrous scaffolds augmented cardiomyogenic differentiation of MSCs through an epigenetic mechanism, involving histone deacetylase SIRT6 [110].

Application of methods that allow 3D printing in a support bath provide a platform for the patterning of soft bioinks into complex, well-defined structures. These technologies use an extrusion 3D printer that deposits material, not on a flat surface in air, but into a bath that suspends the printed material, preventing settling and collapse [111].

Patient-specific, vascularized cardiac constructs of clinically relevant size have been obtained through 3D printing within an alginate–xanthan gum supporting bath, using cells and ECM material derived entirely from the patient’s fatty tissue as bio inks [112].

Cells were reprogrammed into iPCS cells and differentiated to cardiomyocytes and endothelial cells. A vascular network was printed using a sacrificial gelatin ink. The cardiac tissue structure was designed by CAD software that used the patient’s anatomical data from the images obtained by a computed tomography. A cellularized, perfusable, and miniaturized heart (20 mm height; 14 mm diameter) was also printed. However, the printed heart was not able to pump blood and the printed blood vessel network was of limited extension.

Application of the optimized FRESH technology (printing by embedding the bioink in a thermally reversible supportive hydrogel) during printing allowed fabrication of high-resolution 3D anisotropic cardiac structures, such as collagen beating ventricles (5.7 mm diameter, 8 mm height), populated with human cardiomyocytes [113].

Overall, these studies highlight the important effect of nanoscale and microscale topological cues on cell behaviors in cardiac TE. Since both cardiac and skeletal muscle tissues are sensitive to electrical stimulation, research was recently focused on scaffolds based on conductive polymers. Therefore, in the next session we discuss several effects of conductive scaffolds on cardiac or skeletal muscle cells

### 5.2. Conductive Micro/Nano-Structured Scaffolds for Striated Musce Regeneration

Conductive polymers (CPs), such as polyaniline (PANI), polypyrrole (PPy) and poly(3,4-ethylenedioxythiophene)/PEDOT, are smart materials that are usually doped with a p-type or n-type dopant to create native polarons or bipolarons within the polymer structure, thus imparting conductivity [114]. With good processability when blended with inert biomaterials and control over scaffold design niches, an electroconductive microenvironment could be offered in the form of a conductive scaffold to the cultured cells in the context of regenerating cardiac, nerve and skeletal muscle tissues. 

To mimic the striated skeletal muscle tissue architecture, nanoyarns (diameter = 25 µm) made of PANI blended with PCL and silk fibroin were fabricated via electrospinning. Photosensitive poly(ethylene glycol)-co-poly(glycerol sebacate) square block was used as the shell with this nanoyarn in the middle as the core. This core–shell geometry led to an excellent alignment of the cultured C2C12 cells that resulted in the production of myotubes seven days post culture [115]. Electrospinning is a versatile technique to mimic the fibrous nano-bioarchitecture of the extracellular matrix. The pore size of aniline pentamer-modified polyurethane/PCL electrospun membranes ranged from 5 to 150 µm, with an average diameter of 20 µm and 75–80% porosity. These porous membranes significantly enhanced the cardiac specific markers, such as actn4, Cx43, and cardiac troponin T2 (cTnT2) when cultured with neonatal rat cardiomyocytes. Conversely, cells cultured on non-conductive PCL membranes could not develop better contractile phenotypes, justified by the relatively reduced expression of actn4 and cTnT2 [116].

It was observed that decreasing the fiber diameter from 650 µm to around 110 µm led to smooth muscle-like morphology rich in microfilaments when gelatin/PANI membranes were cultured with H9c2 cells. On pristine gelatin and conductive membranes with thicker fibers, cells were randomly oriented with less directionality [117].

Magnetic-field-assisted electrospinning of PCL/PANI led to conductive membranes with super-aligned fibers (~0.3 µm average diameter). Cultured C2C12 cells on these membranes generated myotubes with high maturation and fusion indices five days post-culture. The myotubes also demonstrated significant expression of myosin heavy chain (MHC) markers compared with cells cultured on membranes with randomly aligned fibers [118]. Nano-patterned silk fibroin/PPy conductive scaffolds with groove–ridge topography allowed cells to develop near physiological sarcomeric lengths. Human pluripotent cell-derived cardiomyocytes directionally aligned themselves along narrow grooves (0.8 µm wide) and, after three weeks of culturing, the cells demonstrated a sarcomeric length of 1.86 µm [119], close to the average sarcomeric length of human cardiomyocytes in a relaxed state of about 2.2 µm [120]. Likewise, microgroove–ridges (3.21 µm wide) created on PEG/PEDOT hydrogel led to the myogenesis of the cultured C2C12 myoblasts. Seven days post culture, cells generated myotubes that aligned themselves within ±10° of the groove direction and demonstrated twice the expression of MHC and myogenin, while the cells cultured on non-patterned hydrogels had random orientation, with low expression of MHC and myogenin [121]. These studies elucidate the significance of micro/nano-architecture of the scaffold to develop actual tissue like constructs in vitro with striated morphology. 

To mimic the microfibrous architecture of the cardiac ECM, the electrohydrodynamic printing technique was utilized to fabricate multiscale scaffolds with four layers, with fibers aligned along 0°, 45°, 90° and 135° for the 1st, 2nd, 3rd, and 4th layer, respectively. Continuous repetition of these four layers made a 3D-PCL/PEDOT:PSS-PEO conductive scaffold to mimic the hierarchical structure of the myocardium. In the cardiac ECM, the diameter of the epimysial fibers is around 8.0 µm, while that of endomysial fibers is around 0.5 µm. The diameter of the printed PCL fibers was around 9.5 µm and that of PEDOT:PSS-PEO fibers 0.47 µm. Cultured H9c2 cells demonstrated enhanced expression of α-actinin and Cx43 eight days post-culture, and primary cardiomyocytes started to beat synchronously with a 1.46-fold increase in beating frequency [122].

### 5.3. Micro/Nano-Structured Scaffold for Cartilage Regeneration 

Cartilage is a flexible connective tissue that covers and protects the ends of long bones at the joints and nerves and is a structural component of various body parts, such as the rib cage, the ear, the nose, the bronchial tubes, and the intervertebral discs. Cartilage is composed of a low number of specialized cells called chondrocytes and a large amount of ECM which consists of collagen and elastin fibers embedded in a proteoglycan-based ground. The proteoglycans in the cartilage matrix are highly negatively charged, thereby attracting a large volume of water into the cartilage tissue [123]. Compared with other connective tissues, cartilage has limited repair capacity due to its avascular, aneural structure, low cell density, and because it prevents access of progenitor cells to the injury site. Consequently, cartilage repair represents one of the most important challenges in musculoskeletal medicine, especially for treating injury of articular cartilage [124]. 

TE and material-based technologies can offer a promising strategy for cartilage repair and regeneration. Chondrocytes, mesenchymal stem cells, and genetically modified cells, have all been considered as cell sources for cartilage repair [125].

While chondrocytes are safer for clinical applications, mesenchymal stem cells have the potential to differentiate into both cartilage and subchondral bone cells. 

Collagen scaffolds grown with autologous chondrocytes underwent clinical trials and commercial collagen type I/III scaffolds were produced (e.g., MATC or MACI). The collagen-based scaffolds generally showed improvement in clinical and histological outcomes compared with the control group. Nonetheless, long-term performance of these collagen scaffolds may be compromised by weak mechanical properties and a lack of proper architecture and limited chondrogenic capacity [126].

In order to improve scaffold performance, a large number of natural or synthetic biomaterials were used to create scaffolds to mimic the native structure of cartilage tissue. Several studies focused on the use of the decellularized matrix for the construction of the scaffold.

In a recent study, scaffolds with defined cartilage architecture were fabricated using engraved decellularized cartilage, derived through a CO2 laser of human articular cartilage and then compared to commercial collagen type I/III scaffolds [127]. These scaffolds with engraved grid-patterns, exhibited the ability to guide the new collagen fibers towards a vertical alignment, and improve chondrocyte and mesenchymal stromal cell differentiation, as demonstrated in vitro and in vivo studies.

Porous structures of scaffolds are also extremely important for cell adhesion, spatial distribution, and cartilage tissue regeneration. PLLA porous scaffolds with 100 and 200 µm pore sizes, were fabricated via the TIPS method and seeded with articular and nasoseptal chondrocytes [128]. Most of both chondrocyte types survived on both scaffolds for the whole culture period (7 and 14 days). However, the analysis of the chondrocyte scaffold constructs indicated that the smaller pore dimensions promoted the differentiation of the chondrocytes, justified by an increased gene expression of ECM proteins and cartilage marker (type II, I collagen, aggrecan, SOX9), compared with the larger pore size. 

Conversely, 3D PCL scaffolds [129] and collagen–hyaluronic acid scaffolds [130], with the largest pore size being 300 µm, were superior in supporting the chondrogenic differentiation of MSCs, suggesting that positive influence of pore size depends on cell type.

In addition, the combination of the pore sizes with their geometric shapes can have a great influence on the mechanical properties of the scaffold and cellular behaviors. Scaffold geometries were built by the extrusion-based bioprinting (EBB) technique using two different biomaterials: 1,4-butanediol thermoplastic polyurethane (b-TPUe) and polycaprolactone (PCL) [131]. Three different geometrical patterns were included: hexagonal, square, and triangular; each one was printed with three different pore sizes (PS): 1, 1.5 and 2 mm. Among the nine scaffolds analyzed, the thermoplastic polymeric scaffold with triangular pores and 1.5 mm size showed the optimal conditions for the mechanical properties of the scaffold, which in turn increased MSC adhesion and proliferation. 

Composite or hybrid hydrogel-based scaffolds were intensively studied for cartilage TE. The hydrogel matrix can be reinforced by the incorporation of organic or inorganic nanomaterials, which increase the surface reactivity, mechanical proprieties, and release of loaded bioactive agents [125].

Among inorganic nanomaterials, graphene can adsorb a plethora of biological molecules, thus offering high potential as a delivery carrier when incorporated within natural [132] or synthetic [72] -based hydrogel for TE cartilage applications.

In another study, incorporation of graphene oxide nanosheets (GO) within a poly-D, l-lactic acid/polyethylene glycol (PDLLA) hydrogel enhanced mechanical strength, and supported long-term, sustained release of TGF-β3, which in turn improved the chondrogenic differentiation and cartilage matrix production of encapsulated human MSCs [72]. 

This PDLLA hydrogel was fabricated by using visible-light-based projection stereolithography (VL-PSL), allowing for precision fitting of the anatomy of the damaged cartilage. To meet the regenerative requirements of the heterogeneous and layered structure of native articular cartilage tissue, a number of multilayered biomimetic scaffolds were studied.

In a recent study, the combination of cryo-printing and electrospinning has allowed the fabrication of PCL multizone scaffolds, possessing the high porosity and fiber orientation of the native cartilage [133].

The scaffolds exhibited three distinctive zones to simulate the different zonal structure of the articular cartilage. The bottom layer of the scaffold consisted of a cryo-printed helix which represented the deep zone of the cartilage. Electrospinning was used to deposit orientated and highly aligned fibers onto the bottom layer, which represented the middle and superficial zone of the native cartilage, respectively. These multizone scaffolds supported chondrocyte adherence, growth, and differentiation over a 5-week culture period.

Liu et al. developed a multi-layered osteochondral scaffold through extrusion 3D printing [134]. The scaffold included three layers: a 15% GelMA hydrogel for cartilage on the top layer; a combination of 20% GelMA and 3% nanohydroxyapatite hydrogel for the interfacial layer; and a 30% GelMA 3% nanohydroxyapatite hydrogel for the subchondral bone at the bottom layer. The construct was showed to repair an osteochondral defect in a rabbit knee within 3 months, supporting cartilage and subchondral bone neoformation. 

### 5.4. Micro/Nano-Structured Scaffolds for Bone Regeneration

Bone is a hard type of connective tissue that provides the structural framework for the human body, protection of vital organs, and a stable base for muscle and joint function. Bone also plays an important physiologic role in supporting hematopoietic and mineral homeostasis activities [135]. Human bone has an intrinsic regenerative capacity due to the synergistic actions of mesenchymal cells, osteogenic cells, and cells of the immune system. However, bone repair can fail in degenerative bone diseases or after extensive trauma, and tissue engineering approaches can play a key role in addressing these challenges [136].

Native ECM bone is a multi-component composite material consisting of a hard inorganic phase (minerals, mostly hydroxyapatite, a calcium phosphate ceramic) which provide load-bearing strength and firmness; an elastic, organic network (mainly type I collagen), which imparts flexibility; and a cellular component [137,138]. The cellular component of the bone includes osteoblasts, osteoclasts, osteocytes, and bone-lining cells, embedded in the ECM [139].

The excellent mechanics of native bone are closely associated with its hierarchical structure from the macro to the nano scale, in which the mineralized collagen fibril (diameter ~100 nm) represents the characteristic unit [20,140]. On the nanoscale, hydroxyapatite (HA) crystals are periodically deposited within the gaps (of approximately 30 nm) between collagen molecules inside the collagen fibrils. The collagen fibers (diameter ~1 µm) are composed of bundles of mineralized collagen fibrils (diameter ~100 nm) to form lamellae with different collagen fiber patterns [20] (Figure 2). 

In the cortical bone, collagen fibers are organized regularly to form lamellae densely packed in cylindrical structures, while in cancellous bone, collagen fibers are irregularly arranged to form a porous trabecular tissue mesh [141]. Cortical bone has a porosity of 5% to 15%, whereas the porosity of trabecular bone ranges from 40% to 95%. The internal arrangement and quantity of compact bone and cancellous bone vary according to the type of bone (e.g., long, short, flat bones) [142]. 

To mimic the native composition of bone many composite materials were derived from a combination of inorganic materials (e.g., ceramics, such as HA, calcium phosphate bone cements (CPS), bioglass (BG), glass-ceramics) with natural (e.g., collagen, hyaluronic acid, silk fibroin) or synthetic polymers (e.g., PLLA, PCL, PLGA) [19]. The micro and nanoscale features of natural bone such as porosity, surface topography and fiber alignment, should be rethought to design scaffolds able to stimulate effective tissue growth [137]. 

An interconnected 3D pore structure is a key requirement for bone tissue scaffolds that should allow proper cell accommodation, migration of osteoprogenitors and immune cells, vascularization and innervation. The optimal size of the micropores to facilitate cell bone infiltration and attachment is believed to be in the range of 50–150 µm [30]. However, macropores (100–600 µm) allow better integration with the host bone tissue, vascularization, and new bone formation [20]. Many approaches have been employed to fabricate scaffolds with different pore parameters. 

The TIPS technique supported by the salt-leaching process was used to study the influence of HA content in PLLA /HA scaffolds on pore structure, density, porosity, mechanical properties, and osteoblast proliferation [58]. A greater HA content within the scaffold (up to 75wt%) was related to higher roughness of the pore wall surfaces, porosity (96–98%), and the proliferation rate of osteoblast cells. A more recent study by the same research group showed that the apatite whiskers (HAP) completely covered the pore wall surface of PCL/HAP scaffolds, determining a higher surface roughness [143]. These scaffolds exhibited high porosity (approximately 90%) and heterogeneous pores of two orders of size: one was bigger, with a diameter of up to 600 µm, and the second type of pores which was located in the walls of the micropores, had a diameter up to 50 µm. L-lysine modification of hydroxyapatite could improve the bioactivity of PCL /HAP scaffolds promoting osteoblast proliferation and differentiation, as well as enhancing the mechanical properties and surface roughness of wall pores [143]. Compared with smooth surfaces, a rough topography has been suggested to more effectively mimic the mineralized interface encountered by cells adhering to the native bone ECM [19].

Porous collagen scaffolds with aligned and homogeneous pore with a size of 89  ±  15 µm, were produced by controlled directional freezing and freeze-drying of a 1.5% (wt/wt) collagen dispersion. The channel-like pore architecture of these scaffolds induced progenitor cell recruitment, ECM alignment, and finally, a highly organized endochondral ossification process in rat femoral bone defects [144]. The nano/micro cues involved in the architecture of bone scaffolds have been perfectly reproduced into porous scaffolds by Liu and colleagues. In this study, hierarchical intrafibrillarly mineralized collagen with a bone-like staggered nanointerface was fabricated using self-assembly and thermodynamic control methodologies. The resultant scaffold showed a bone-like hierarchical architecture which induced neo-bone formation by promoting M2 macrophage polarization and host MSC recruitment in critical-sized bone defects [145].

In another recent study, porous β-tricalcium phosphate (β-TCP) scaffolds were built by combining the digital light processing (DLP) printing technique and the in situ growth crystal process. These bioceramic scaffolds with macro- and micropores were produced by DLP printing. Afterwards, the in situ growth crystal process was used to produce the micro/nano surface topography. The micro/nano structured scaffold facilitated the proliferation and differentiation of rat bone MSCs (rBMSCs) and displayed remarkable skull bone regeneration capacity in a rat model [146]. Native bone tissue presents a structural gradient which can be identified in a radial direction in long bones, and in an axial direction in flat bones, due to the variation in bone density and pore parameters from the cancellous bone to the cortical bone [146]. Taking inspiration from the natural bone structure, Di Luca et al. fabricated 3D PCL scaffolds presenting an axial gradient in pore size and total porosity by using the “Fused deposition modelling” (FDM) method. This scaffold improved the osteogenic differentiation of hMSCs according to the pore size with greater effect on cells residing in larger pores [147].

Three-dimensional printing technology was used to produce two type of 3D bioceramic scaffolds with different internal architectures, displaying either a designed porosity gradient or a constant pore distribution. These hydrogel–ceramic composites were made of methacrylated-oligocaprolactone-poloxamer and low-temperature self-setting calcium-phosphates. The scaffolds encased within a non-porous PCL chamber were implanted in equine orthotopic bone defects. After 7 months, both types of scaffold showed a capacity to support new bone formation which, however, was greater in structures with constant porosity. In addition, replacing PCL with non-degradable materials was recommended by authors [148]. Bone ECM presents a micro/nano fibrous component which guides cell alignment and thus, micro and nanofibers are often produced by electrospinning and other techniques to improve osteogenesis [149]. A 3D honeycomb-shaped scaffold micro-nanostructure was developed by Nedjari et al. To manufacture this scaffold, honeycomb-arranged nanofibers consisting of poly (L-lactide ε-caprolactone) and fibrinogen were produced by electrospinning, using a honeycomb-shaped collector produced by photolithography on silicon wafers. The nanofibers with the honeycomb architecture induced cell osteogenic differentiation, as justified by the superior cellular deposition of phosphate and calcium, and the increased expression of the relevant gene marker of ALP activity [150].

A hybrid Silica–Silk Fibroin aerogel scaffold with honeycomb-shaped micromorphology was developed by Maleky et al. through a novel aqueous-based sol–gel process, followed by unidirectional freeze-casting, and supercritical drying approaches [151]. The resulting aerogel showed high Young’s modulus (~4–7 MPa), high porosity (91–94%), and hierarchically organized porous and microstructural alignment (anisotropy) in varied length scales. The microstructure of the Silica–Silk Fibroin aerogel promoted osteoblast cell attachment, growth, and proliferation and in vivo bone formation, when implanted in the rat bone defect. 

## 6. Challenges and Conclusions

Over the last two decades, a plethora of studies produced an ever-growing list of micro/nano structured biomaterials for tissue regeneration. The range and degree of biomaterial sophistication also dramatically increased as more knowledge has accumulated through materials science, cell biology, and tissue engineering. Innovative techniques and their combination have made it possible to rethink the micro and nanoscale architectural features of the scaffold with extraordinary precision. Advances in manufacturing techniques have enabled the creation of scaffolds containing information increasingly similar to native ECMs. Additive manufacturing and particularly 3D-printing processes have emerged as the most promising technologies to fabricate patient-specific scaffolds and allow for the easy modification of multiple structural parameters.

Despite micro-/nanoscale biomaterials hold great promise in clinical medicine, their clinical translation has been relatively slow. Although tremendous progress has been achieved in some field such as cartilage TE and some scaffolds are already reaching the market, issues for in vitro reconstruction of well-structured and functional complex tissues with low regenerative potential such as myocardium and bone, are still prevalent. 

One of the problems is that even with the existing advanced micro- and nanofabrication techniques, it is difficult and challenging to prepare highly ordered micro and nanoscale features over a large area for constructs of clinically relevant size. Another challenge is generating the billions of cells required to 3D-bioprint large tissues. To obtain a large number of tissue-specific differentiated cells, iPSCs, derived from adult somatic cells, hold promise for patient-specific therapy and can differentiate into each cell type. However, their culture and differentiation are expensive. In addition, iPSCs still raise concerns about the persistence of pluripotent cells after differentiation and their inherent genetic instability upon prolonged culturing and the consequent risk of teratoma formation [152]. Nowadays, advanced micro- and nanofabrication techniques such as 3D bioprinting have allowed to build constructs that start to rethink the structural, mechanical, and biological properties of native tissue. However, the level of resolution that is required in order to achieve fully functional constructs, remains unknown. On the other hand, imitating the complexity of human tissues is not easy. 

Indeed, native tissues and organs are complex ensembles of multiple cell types embedded in an extracellular matrix in which its components are often hierarchically organized to form highly defined structures such as the intricate vascular and neural networks.

The reconstruction of a functional vascular network within engineered tissues is one of the major limitations hampering clinical translation. Despite the progress in engineering vascular channels within tissues, the construction of a multiscale vessel containing both microscale capillaries and mesoscale vessels remains a grand challenge [153]. Three- dimensional printing techniques enable the organization of vascular cells to form only mesoscale vessels. Three-dimensional lithography allows the fabrication of channels as small as 3 µm in diameter; however, it is challenging to flow endothelial cells through these microchannels without clogging them [153]. Many studies are exploring the development of adequate procedures to create functional vascularization. For example, it was recently reported that microscale capillaries can be formed by the self-organization of endothelial cells seeded both into patterned microchannels and the surrounding collagen matrix [154]. Nevertheless, further efforts are still needed to produce a functional vascular network and capillaries.

Moreover, human organs are highly organized dynamic entities, which maintain homeostasis, transfer information, generate force, and adapt to changes in the environment.

Cells and the surrounding microenvironment dynamically interact with each other during cell growth and tissue regeneration. ECM properties, including stiffness, topography and chemical composition, spatially and temporally influence cell fate and function. Although the traditional scaffolds prepared with static biomaterials can regulate cell behaviors, they fail to precisely mimic the dynamic microenvironment of the natural tissues. 

Recently, smart biomaterials attracted increasing attention due to their capacity to respond to external stimuli, such as heat, pH, light, etc. 

Among these smart materials, shape-memory polymers endow the creation of biomimetic micro/nano-constructs which dynamically change their architecture in response to their microenvironment [155]. These shape-memory polymer-based constructs have the capacity to regulate cell behaviors and prompt tissue growth in a spatio–temporal way, thereby mimicking dynamic changes in the ECM structure, both ‘in vitro’ [156] and ‘in vivo’ [157]. In addition, these responsive materials can be implanted through minimally invasive surgery and fill irregular defects.

Smart/dynamically responsive biomaterials can be stamped using 4D printing, i.e. “the creation of objects which alter their shape when removed from a 3D printer” [157]. Dynamically responsive materials and 4D printing offer the possibility of mimicking not only the chemical, physical and architectonic characteristics of living tissues but also the ECM dynamic environment. Four-dimensional bioprinting promotes dynamic, structural, and cellular changes of tissue over time [158]. The responsive materials and 4D technology open an unprecedented scenario in the field of regenerative medicine and tissue engineering that promises great progress, such as the printing of whole functional organs. This will help overcome the static nature of 3D bioprinting and create tissue-like models that resemble those found in nature.
